# Coxa pedis: can calcaneal pronation angle be considered a predictive sign of medial plantar arch overload?

**DOI:** 10.1007/s11547-024-01815-w

**Published:** 2024-04-24

**Authors:** Marco Calvi, Dario Grasso, Giulia Sollami, Silvia Lamantea, Tonia Gatta, Aroa Gnesutta, Raffaele Novario, Alberto Aliprandi, Eugenio Annibale Genovese

**Affiliations:** 1 Department of Diagnostic and Interventional Radiology, ASST-Settelaghi, Ospedale di Circolo e Fondazione Macchi, Varese, Italy; 2 University of Insubria: Università Degli Studi Dell’Insubria, Varese, Italy; 3 Section of Radiology, Department of Biomedicine, Neuroscience and Advanced Diagnostics, University of Palermo, Palermo, Italy; 4 Medical Clinical Institute Intermedica - Columbus, Milano, Italy; 5 Department of Radiology, Istituti Clinici Zucchi, Monza, Italy

**Keywords:** Coxa pedis, Calcaneal pronation angle, Foot, Posterior tibial tendon, Spring ligament

## Abstract

**Purpose:**

To assess whether a correlation between the calcaneal pronation angle and the presence of internal plantar arch overload signs (such as upper-medial spring ligament lesion, posterior tibial tendon tenosynovitis, etc.) could lead to a better understanding of coxa pedis pathology.

**Material and methods:**

One hundred ankle MRIs of consecutive patients were retrospectively reviewed measuring the calcaneal pronation angle and either the presence or absence of internal plantar arch overload signs. Next, the association of overload signs with increasing pronation angle was evaluated to establish a cut-off point beyond which coxa pedis pathology could be defined.

**Results:**

The tibial-calcaneal angle values in patients with and without effusion proved to be significantly different (*p* < 0.0001). The tibial-calcaneal angle values in patients with and without oedema also demonstrated a significant difference (*p* < 0.0056). Regarding posterior tibial tendon, a significant difference was found between the two groups (*p* < 0.0001). For plantar fascia enthesopathy, the result was borderline significant (*p* < 0.054). A linear correlation was found between the value of pronation angle and the extent of spring ligament injury (*p* < 0.0001). In contrast, no correlation with age was found.

**Conclusion:**

In conclusion, the literature associates medial longitudinal plantar arch overload with posterior tibial tendinopathy and spring ligament complex injuries. Our data show that both injuries are highly correlated with increased calcaneal pronation angle, which could be considered a predictive sign of internal plantar arch overload, prior to the development of the associated signs.

## Introduction

Coxa pedis is the talo-calcaneal-navicular joint and is the distal enarthrosis of the lower limb. It is defined coxa because of the enarthrosic meaning from an anatomical point of view and the analogy to the hip [1].

The talo-navicular joint is an important part of the Chopart joint between the head of the talus, a part of the hindfoot, and the concavity produced by the posterior face of the navicular bone, part of the midfoot. This joint is completed by the talo-navicular ligament, a wide and thin band that covers the extensor tendons, and by the plantar calcaneal-navicular ligament also called spring ligament.

The medial compartment also comprehends the posterior tibial tendon (PTT), another tendinous structure that is part of the capsular-ligamentous and tendon complex. The latter is one of the fundamental dynamic stabilizers of the talo-calcaneal-navicular joint; injuries of this tendon lead to calcaneal pronation. (The calcaneus tends to lean inwards, while the rest of the foot tends to spread outwards.)

The association between PTT tears and injury of the spring ligament complex has been reported in the orthopaedic literature [2].

Diseases affecting the coxa pedis may involve the bony, articular, or internal compartment structures.

Bone pathology may include various types of synostoses between the plantar bones, with manifestations such as tarsal and hindfoot pain, peroneal tendon spasm, pes planus and secondary osteoarthritis [3].

Additional injuries that may affect the bone compartment may manifest as stress fractures, particularly fatigue and insufficiency fractures [4].

Joint pathology develops as osteoarthritis and sinus tarsi syndrome, with development of pain and tenderness of the lateral side of the hindfoot, between the ankle and the heel, feeling of instability and pain on palpation [5].

Lastly, injuries of the medial complex may involve the spring ligament and the posterior tibial tendon [6].

Given the anatomical complexity of the joint, the broad spectrum of pathologies that can affect it, and due to the non-specific symptomatology, which can mimic disorders of other compartments, an accurate knowledge of coxa pedis’ anatomy and the evaluation of factors that can potentially lead to injuries of this area is particularly important.

The hindfoot structures play a crucial role in absorbing and transforming forces during both passive and active phases of gait. During the passive phase of gait, the hindfoot acts as a shock absorber, dissipating the forces generated from ground contact and distributing them throughout the foot and lower limb. This process is essential in reducing impact-related injuries and ensuring smooth transitions between gait phases. In the active phase, the hindfoot transforms stored energy into propulsive force, aiding in forward propulsion and locomotion. Structures such as the talo-calcaneal-navicular joint, talo-navicular ligament, and posterior tibial tendon play integral roles in these mechanisms, providing stability, support, and dynamic control. Therefore, a comprehensive understanding of hindfoot anatomy and function is paramount in assessing and managing conditions affecting gait mechanics and lower limb biomechanics [2, 7–9].

The purpose of our study is to investigate the relationship between the calcaneal pronation angle and injuries resulting from internal plantar arch overload. In particular, we aim to establish whether there is any correlation between the pronation angle and the presence of lesions of the SLC and PTT.

## Material and methods

### Patients

One hundred ankle MRIs of consecutive patients were retrospectively evaluated, progressively performed from February 2022 to September 2022. The main clinical issue was persistent ankle pain. The following exclusion criteria were applied to the initial sample: no surgery nor arthroscopy prior to the examination, no recent traumatic events, age between 18 and 60 years, no signs of advanced arthrosis and absence of inflammatory arthropathies.

### MRI technique

All MRI examinations were performed using a 1.5 T unit (Achieva XR, Philips). From each patient, written informed consent was obtained.

Each patient was positioned supine with both lower limbs extended and the feet resting on a soft support to achieve approximately 55 to 65 degrees of dorsiflexion. The image acquisition protocol is summarized in Table 1. No restraints were used to immobilize the ankles during the examination. MRI images were evaluated using a dedicated workstation.

**Table 1:** MRI parameters: image acquisition protocol

	TSE T1	SPAIR	DP fat-sat TSE T2
	Sag	Sag/Cor	Axial
TR (time to repeat) (ms)	400	3200	4330
TE (time to echo) (ms)	14	32	81
Matrix	240 × 320	240 × 320	358 × 448
Thickness (mm)	3	3	3
Number of signal averages (NSA)	1	1	2

### Image analysis

**Fig. 1:**
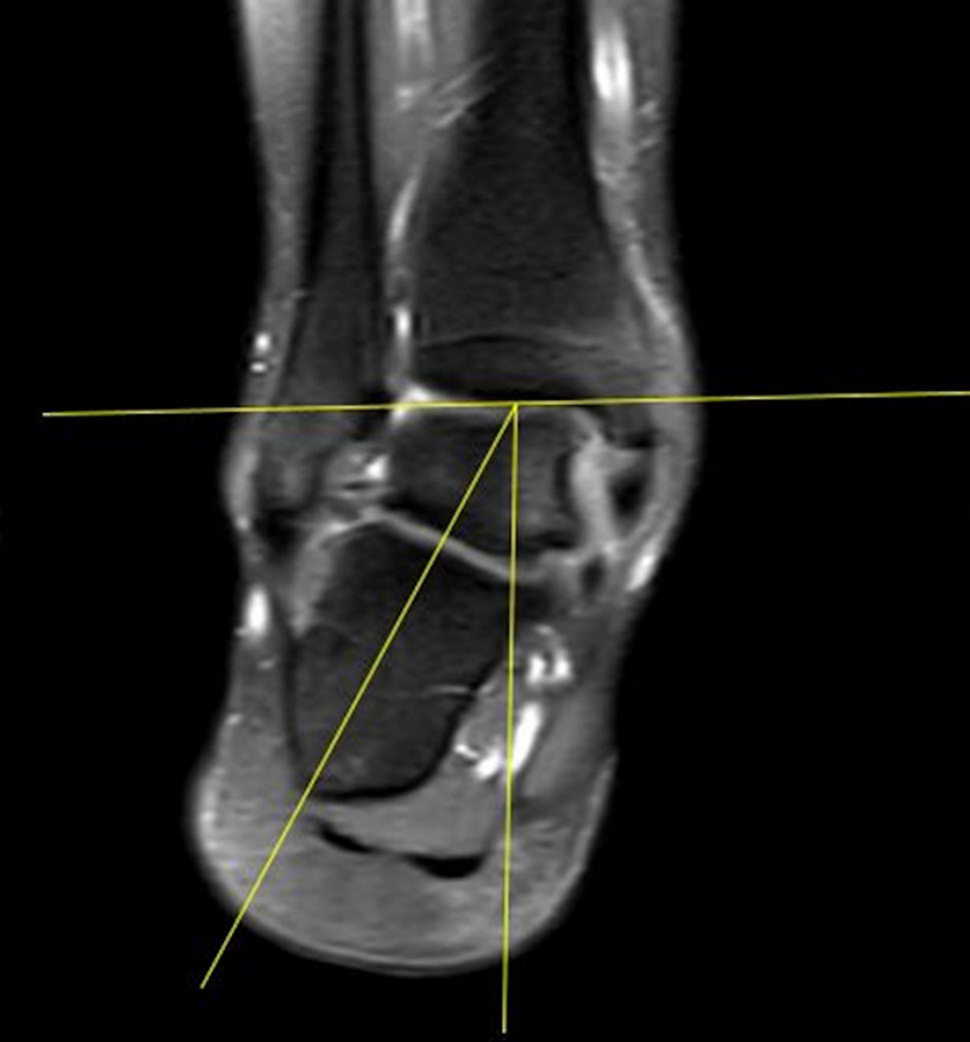
Coronal SPAIR sequences. We measured the tibial-calcaneal angle as the angle between a line perpendicular to the transverse axis of the distal tibia and a line parallel to the medial calcaneal contour

All images were reviewed using a Picture Archiving and Communications System (PACS) by two radiologists, one with 4 years of experience and specific training in MSK pathology and the other with more than 20 years of experience in reading musculoskeletal MRIs, both blind to the original reports. In all examinations, the pronation angle of the calcaneus was measured in the coronal plane at the level of the posterior subastragalic joint. A tangential line to the deepest point of the tibial articular surface was marked, a line perpendicular to this and a line longitudinal to the axis of the calcaneus. The angle was measured between the latter two lines (Fig. 1).

**Fig. 2:**
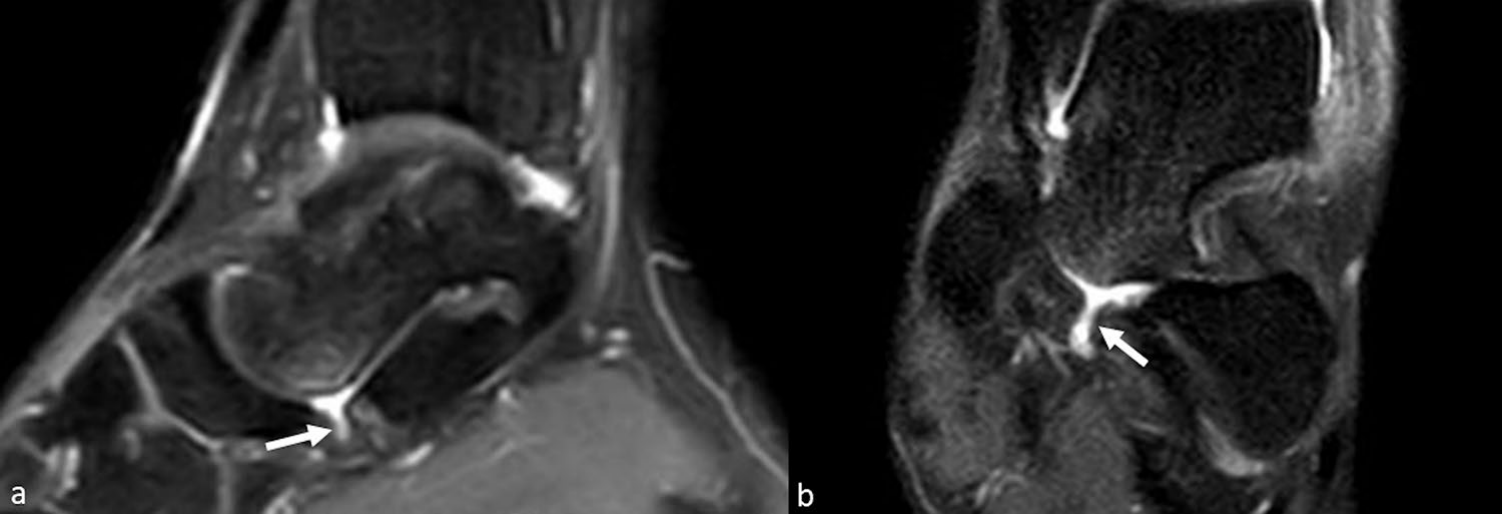
Sagittal (**a**) and coronal (**b**) SPAIR sequence demonstrates fluid in talo-calcaneal recess (white arrows)

**Fig. 3:**
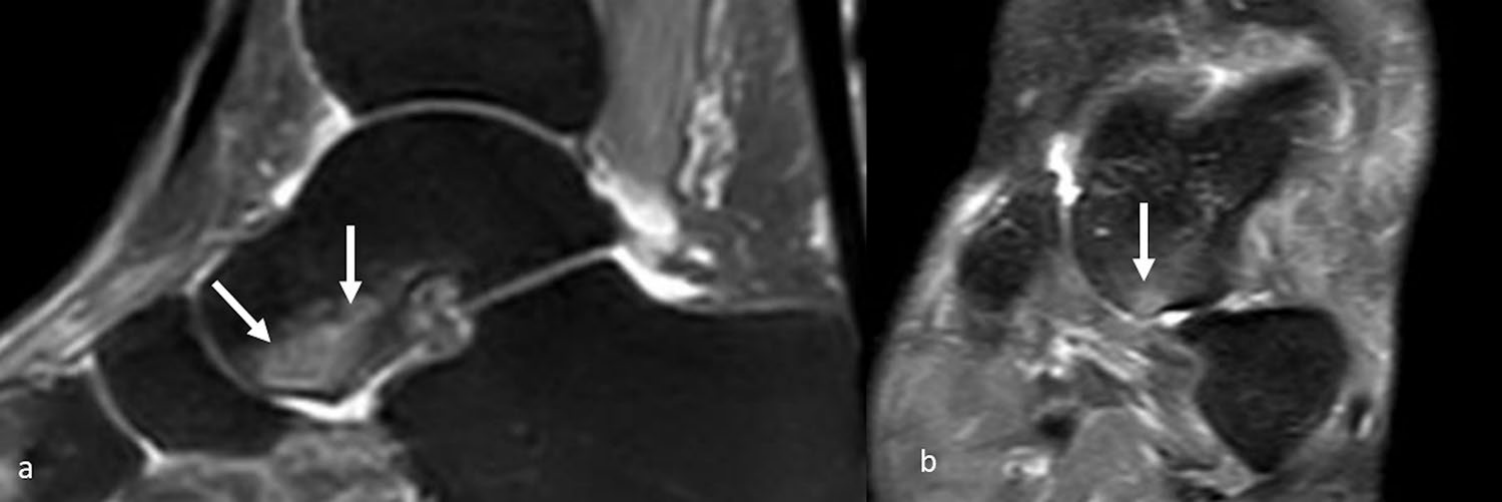
Sagittal (**a**) and coronal (**b**) SPAIR sequences demonstrate trabecular oedema of the talus head (white arrows)

**Fig. 4:**
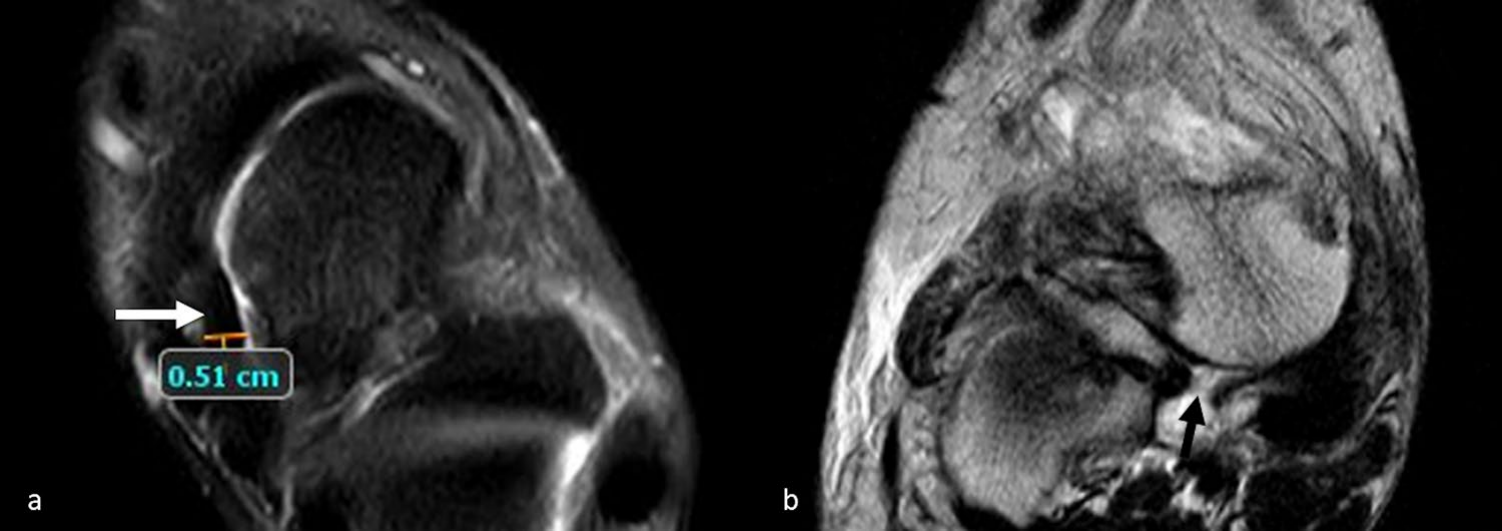
Axial fat-sat proton density-weighted sequence **a** shows a group 1 injury of the upper-medial spring ligament bundle as an increased thickness or a heterogeneous signal in the ligament (white arrow). Axial TSE T2-weighted sequence **b** shows complete lesion (type C) of the upper-medial bundle of the spring ligament (black arrow)

**Fig. 5:**
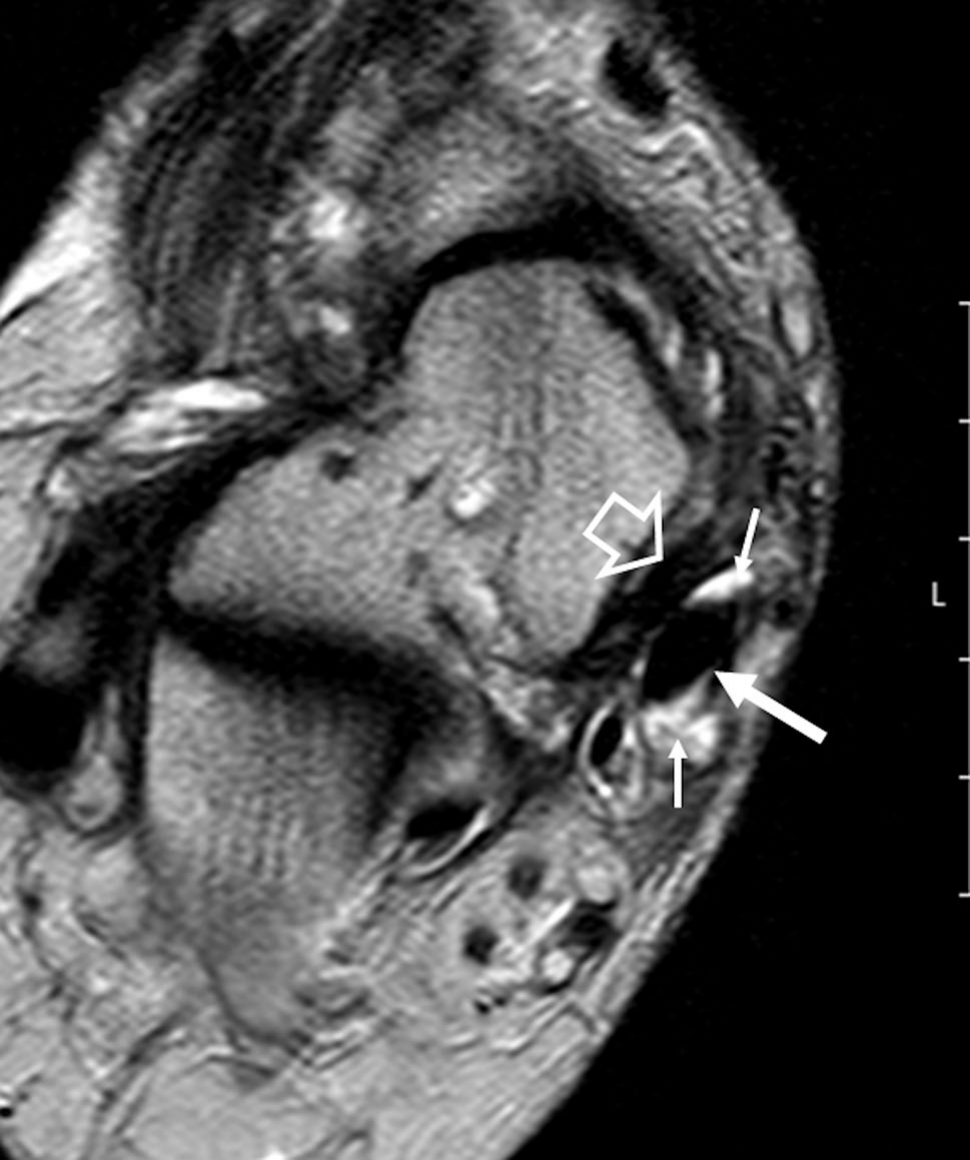
Axial TSE T2 weighted sequence shows posterior tibial tendon injury as marked thickening (large white arrow) and effusion in synovial sheath (thin white arrows). In the same image, type A lesion of the upper-medial spring ligament bundle is observed (empty arrowhead)

The following parameters were analysed:

- Calcaneal pronation angle (CPA); measured as a quantitative variable in coronal SPAIR sequences (Fig. 1). It is defined as the angle between a line perpendicular to the transverse axis of the distal tibia and a line parallel to the medial calcaneal contour. The study by Buck et al. [10] demonstrated that measurement of the tibio-calcaneal angle using the medial calcaneal contour as a landmark produced the highest accuracy, achieving a sensitivity of 86% and a specificity of 75%.
- Effusion in the scapho-calcaneal space (SCSF); evaluated as a binary variable in the T2-weighted sequences acquired in the axial, coronal and sagittal plane as illustrated in Fig. 2.
- Trabecular oedema of the talus head (THE); assessed as a binary variable in axial FSE T2-weighted sequences and SPAIR sagittal and coronal sequences (Fig. 3).
- Injury of the upper-medial spring ligament bundle (UML); classified into three subgroups according to injury extent (group 1—thickening; group 2—thinning or irregular signal intensity; and group 3—lesion) [11]. Spring ligament abnormality was noted when the UML was thickened to more than 5 mm, thinned, or showed heterogeneous signal in the ligament [12]. Thickness was primarily evaluated in the axial sequences (Fig. 4).
- Posterior tibial tendon (PTT) injury; assessed as a binary variable in the axial fat-sat images and coronal SPAIR sequences (Fig. 5). It was considered positive if tendinosis (marked thickening and increased signal within the tendon), partial tear (thinning or a split tear of the tendon) or complete tear (disruption of the tendon) was present.
- Plantar fascia enthesopathy (PFE); assessed as a binary variable in FSE T1 and SPAIR sequences acquired in the sagittal plane. It was diagnosed if one or more of the following signs was present: thickening of the plantar fascia, presence of intrasubstance lesions and presence of bone marrow oedema at the enthesis [13].

### Statistical analyses

The statistical study was conducted by dividing the tibio-calcaneal angle values in accordance with the previously listed binary variables (effusion, oedema, tenosynovitis and enthesopathy). Subsequently, the distribution of each data set was evaluated so that the appropriate test for comparison could be chosen. In case of normal distribution, Student's T test was applied; otherwise, Mann–Whitney test was applied. Next, the correlation degree between calcaneal angle and the extent of spring ligament injury was evaluated using linear regression. A possible influence of the patient's age on the amplitude of the angle itself was similarly investigated. In conclusion, for each binary variable, the AUC and the best cut-off value above which a coxa pedis pathology could be suspected were calculated. To obtain the best cut-off value in terms of sensitivity and specificity, the “Youden's J” value was considered. Statistical analyses were performed on the average values of the measurements obtained by each radiologist. Inter-rater reliability (Cohen's K) was calculated for each data set.

Statistical analyses were performed using MedCalc for Windows, version 19.4 (MedCalc Software, Ostend, Belgium).

## Results

From the MRI examinations available in the archive, 100 studies were selected from a sample of patients consisting of 64 males and 35 females with a mean age of approximately 40 years (median 43; min 18—max 60). From the clinical data available, it was possible to deduce that all the selected patients had the examination due to foot pain or impairment during walking. The inter-rater reliability test obtained the following results for each of the binary variables under investigation: SCSF: 0.98; THE: 0.99; UML: 0.88; PTT: 0.98; PFE: 0.95. To calculate the K-factor for the UML category, the values of the first and second groups were merged and set in relation to the third.

**Fig. 6:**
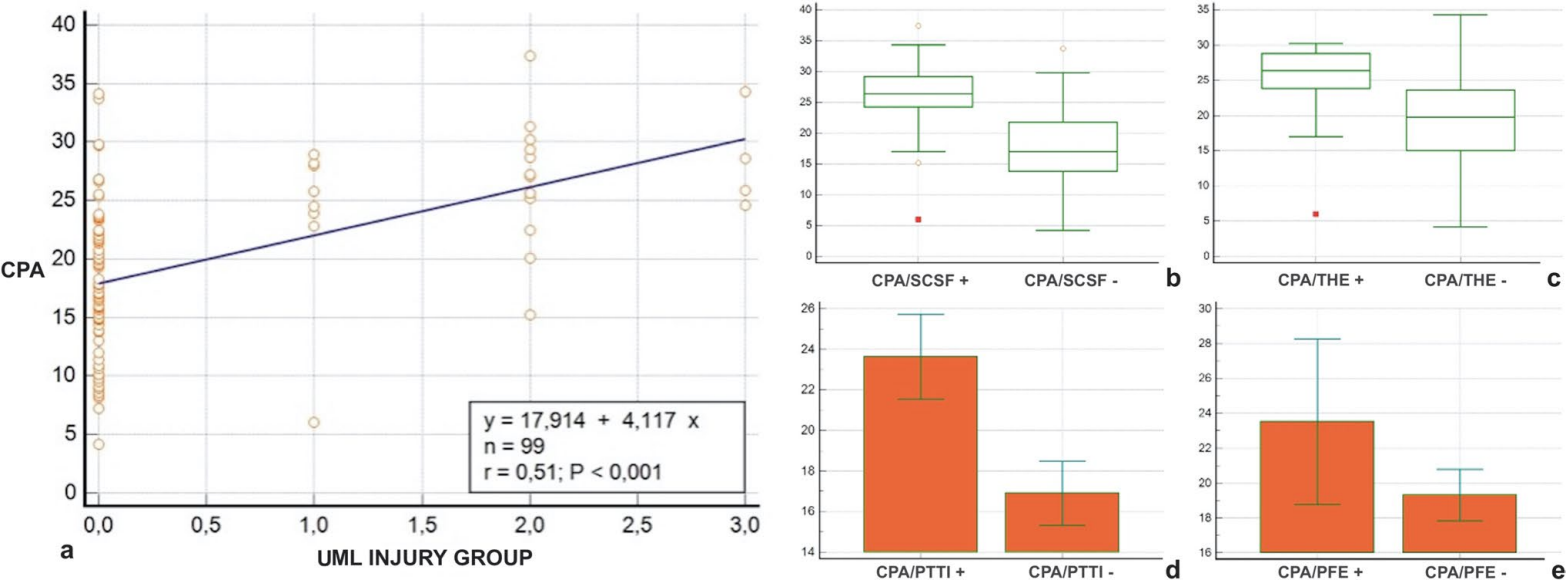
**a** Correlation between calcaneal pronation angle (CPA) and upper-medial spring ligament bundle (UML) lesion (grade 1, 2, 3). **b** Correlation between calcaneal pronation angle (CPA) and scapho-calcaneal space effusion (SCSF); **c** correlation between calcaneal pronation angle (CPA) and talus head oedema (THE); **d** correlation between calcaneal pronation angle (CPA) and posterior tibial tendon injury (PTTI); (**e)** correlation between calcaneal pronation angle (CPA) and plantar fascia enthesopathy (PFE)

The Mann–Whitney test for independent samples was used for the data sets obtained with the binary variables of SCSF and THE. The CPA values in patients with and without SCSF proved to be significantly different (U = 267.50; Z = 5.6; *p* < 0.0001) (Fig. 6a). CPA values in patients with and without THE also demonstrated a significant difference (U = 106; Z = 2.7; *p* < 0.0056) (Fig. 6b).

On the other hand, the data sets obtained with the binary variables of PTT injury and PFE demonstrated a normal distribution, whereby the t-test for independent samples was used. Regarding PTT injury, a significant difference in CPA was found between the two groups (T = − 6.7; CI − 9.26/− 4.19; *p* < 0.0001) (Fig. 6c). For PFE, the result was borderline significant (T = − 4.2; CI − 8.5/− 0.08; *p* < 0.054) (Fig. 6d).

A linear correlation was found between the value of CPA and the extent of UML injury (y = 17.9143 + 4.1171 × *p* < 0.0001) (Fig. 6a). No correlation with age was found.

Regarding the cut-off point to refer for coxa pedis pathology, for each binary variable, the following values were obtained: SCSF (AUC = 0.876; 0.77–0.983; Youden’s J = 23.9, SENS: 0.786; SPEC: 0.914); THE (AUC = 0.764; 0.554–0.973; Youden’s J = 25.2, SENS: 0.75; SPEC: 0.811); PTT lesion (AUC = 0.803; 0.693–0.912; Youden’s J = 23.9, SENS: 0.58; SPEC: 0.94); and PFE (AUC = 0.67; 0.47–0.8; Youden’s J = 25.6, SENS: 0.58; SPEC: 0.826).

## Discussion

In our study, we found that TTP dysfunction may be secondary to SLC injury, which in turn is due to the overloading of the internal plantar arch with increased calcaneal pronation angle.

In fact, there seems to be a correlation between increased heel pronation angle and the occurrence of effusion in the talo-calcaneal joint space, a relationship that is also evident with the onset of talar bone oedema. Pronation angles greater than 24° (CI 23.9–25.2) may cause medial longitudinal plantar arch overload and consequentially SLC injury and PTT dysfunction.

The talo-calcaneal-navicular joint is also identified by the term "Coxa Pedis," a term popularized by Pisani [1], not only in relation to its analogy with the coxo-femoral joint but also for evolutionary and pathogenetic reasons.

The joint's stability is provided by static stabilization systems, such as the peritalar ligaments, calcaneal-navicular ligaments, and the lateral ligament (formed by the inner part of the Chopart's ligament); and dynamic stabilization systems, such as the posterior tibialis muscle tendon and toe long flexor and hallux long flexor muscles tendons [2].

The spring ligament complex (SLC) (also known as the plantar ligament or lower calcaneal-navicular ligament) is a ligament complex that forms the centre of the "acetabulum" of the foot. It is the main static stabilizer of the internal longitudinal plantar arch and consists of upper-medial ligament (UML), mid-plantar oblique ligament (MPO) and inferoplantar longitudinal ligament (IPL) [14]. The UML forms the widest part of the SLC at its inner side [15], deeply envelops the head of the talus and has a close relation with the posterior tibialis muscle tendon (PTT) [14].

MRI studies conducted on corpse anatomical preparations allowed differentiation of all three parts of the SLC allowing comparison between the actual anatomy and the MRI images acquired. The UML was recognizable in all preparations; the MPO in 60 of them; and the IPL in 71.

The average thickness of the UML was measured to be 3.2 mm, best recognized in oblique-transverse or coronal MRI sections. Its physiological signal intensity was defined as intermediate signal in T1-weighted images and low signal in T2-weighted sequences. The average thickness of the MPO was measured to be around 2.8 mm, best seen in transverse sections with a striated-looking physiological signal intensity in both T1- and T2-weighted sequences. The IPL was found to be the thickest among the three (4 mm), best represented in coronal sections, with intermediate signal intensity in T1 and variable signal intensity in T2 [16].

Regarding the loading angles of the foot the tangent line at the sustentaculum tali and the medial contour of the calcaneus in the coronal proved to be the best anatomical landmarks to assess hindfoot varus angle [17].

Classically, hindfoot varus angle is assessed on loading radiographs [18, 19]. In many studies, patients with pathological pronation angles were subsequently evaluated with MRI to analyse the health status of the subastragalic, tibio-peroneal joints and supporting and stabilizing structures.

Although it is incontrovertible that MR is not the method of choice in the evaluation of plantar angles, as it is not performed in weight-bearing, it is equally logical to assume that an angle evaluated as pathological under off-load conditions can only be the same or wider when subjected to load.

In Acquired Adult Flatfoot Deformity (AAFD), there have been described primary injuries, directly involving the PTT, and secondary injuries corresponding to lesions of the associated structures. Rare cases with AAFD secondary to isolated lesions of the SLC have been reported in the literature [20].

The patients enrolled in the present study had a variable clinical presentation related to the cause of flat foot and hindfoot varus. The most frequently documented symptom was internal perimalleolar pain due to PTT dysfunction. [21–23].

The posterior tibial tendon (PTT), thereafter, is the main active stabilizer of the ankle [12, 24, 25], and if it fails, load forces during gait would be transmitted directly to static stabilizers such as the SLC, deltoid ligament, long plantar ligament, and plantar fascia (PF) [12, 24].

In literature, it has been described how a PTT injury produces progressive involvement of the SLC, sinus tarsi, and plantar fascia [2] and that the SLC injury is often consequent to a chronic degenerative process secondary to an insufficient PTT [12, 24].

Therefore, the dysfunction of the latter would cause the loss of dynamic stabilization of the midfoot with increased tensive forces against the UML because of increased hindfoot valgus angle.

For this reason, many authors agree that an SM bundle injury is not possible in the absence of PTT pathology [2, 11] and that hindfoot deformities and SLC changes are often nothing more than a consequence of PTT injury [2, 26, 27]. On the other hand, Yao et al. [12] demonstrated that an abnormality of the SLC has a high association (92%) with an extensive posterior tibial tendon injury.

Yao et al. [12] also described SLC injuries in MRI correlating them with posterior tibial tendon tear without, however, reporting their frequency.

In our series, pronation angle was measured with supine patients looking for any correlation with the presence of primary or secondary injuries. The results obtained could be disputed as they were not acquired under weight-bearing; however, the objection could be considered valid only if the plantar angle values were underestimated, i.e., if injuries with pronation angles within the normal range were present.

A correlation between effusion in the calcaneal-navicular space and heel pronation angle has not been evaluated in the literature. In our study, a CPA greater than 23.9° demonstrated a statistically significant correlation with the presence of SCSF. Our work also acknowledged a correlation between increased CPA and the occurrence of AHE; the latter was present in all patients with CPA greater than 25.2°.

The UML can be reliably assessed by routine MRI, and signs of its injury appear to be highly specific although only moderately sensitive. The best criteria for demonstrating a clinically relevant lesion are increased signal on proton density or T2-weighted sequences with thickening (> 5 mm), thinning (< 2 mm), or partial to complete discontinuity [20].

Statistically, the pathologic ligament is more likely to demonstrate a thickened rather than thinned appearance in T1-weighted images [12]. Chronic ligamentous injuries are less likely to exhibit signal hyperintensity on T2-weighted images and are thus more difficult to diagnose.

All SLC injuries described by Yao et al. [12] were chronic and presented only modest heterogeneity ( ±) and mild signal hyperintensity ( ±) on T2-weighted images. However, patients with chronic SLC insufficiency showed a thickened medial portion of the ligament (mean 6.5 mm, standard deviation 1.3 mm). Based on other investigations, a thickness threshold of 4 mm might be more appropriate [16].

Balen et al. [2] investigated associated injuries in patients with advanced PTT disorders finding a high association with UML abnormalities (92% vs. 28% in the control group with normal PTT).

In their retrospective study, Williams et al. [28] analysed the MRI findings of 13 patients with flexible AAFD with a surgically established UML lesion. The MRI findings demonstrated proximal thickening > 5 mm in 92% and distal thinning < 2 mm in 85%.

The sole finding in cases with surgically established tears is a full-thickness tear in the ligament, found in 79% of cases in our series. When multiple abnormalities are found in the SLC together with a full-thickness tear, the diagnosis of a tear can be made with confidence [11].

In our series, we evaluated three different types of SLC upper-medial bundle abnormalities: we considered ligament thickening greater than 5 mm (group A) signal inhomogeneity (group B) and ligament injury (group C). According to the literature, a correlation between UML injury and TTP abnormalities was demonstrated. In addition, a linear correlation was found between the pronation angle value and the extent of SLC injury (y = 17.9143 + 4.1171 × *p* < 0.0001) (Fig. 6a).

Still in agreement with the literature, a correlation between increased CPA and the presence of PTT lesion was also found in our series (Fig. 6).

However, based on the results obtained, our findings do not coincide with the theory that damage to the SLC is consequential to a dysfunction of the PTT. Indeed, as shown in Table 2 it is evident that PTT dysfunction is present in all cases with upper-medial bundle injury of the SLC, while only one case of isolated PTT lesion was recorded in the absence of SCL injury. The article by Jennings and Christensen refers to experimental research in cadavers highlighting the importance of SLC lesions in the pathogenesis of AAFD [9]. The PTT functions almost identically when the SLC is intact or dissected; however, its dynamic ability to stabilize the medial longitudinal arch appears to be impaired when the SLC is dissected and surgical reconstruction of the PTT does not correct the flatfoot [9].

**Table 2:** Distribution of PTTI and UMF-SLI. Data are presented as average pronation angle (°), mean (max–min)–n° of patients. PTTI: posterior tibial tendon injury; UMF-SLI: spring ligament’ upper-medial fascicle injury

PTTI absent and SLI present (any grade)	28, 28(28–28)–1
PTTI and UMF-SLI absent	17, 16.4 (33.7–8.2)–55
PTTI present/UMF-SLI grade 1	22.7, 25.2(28.9–6)–7
PTTI present/UMF-SLI grade 2	26.6, 27.1(37.4–15.2)–13
PTTI present/UMF-SLI grade 3	29.2, 28.6(34.3–24.6)–4

In line with our theory, several studies have also shown how PTT insufficiency per se is not capable of producing the acquired adult flatfoot deformity (AAFD).

The study by Balen et al. [2] analysed the frequency of SLC and plantar fascia abnormalities evidenced by MRI in a group of patients with advanced PTT lesions.

Balen and Helms report MRI findings correlating the SLC injuries with posterior tibial tendon impairment. They indicate the posterior tibial tendon injury as secondary to the increased functional load due to the SLC injury [2].

PFE has a low association with advanced PTT injury: in the study of Yao et al. [12], only 28% of patients with PTT injury had plantar fascia abnormality. In many studies the appearance of PFE on MRI is described, but the association with PTT insufficiency is rarely reported [12].

In our case series, the relationship between PTT injury and PFE was not found to be statistically significant, nor was there evidence of a relationship between CPA and PFE.

## Limitations

Our study has a few limitations mostly due to the angle measuring methods used. Since we performed MRI scans, we had no information about joint behaviour under load. It is equally true that our results and statements were based on static images, regardless of the in-motion biomechanics of the inner plantar arch. The use of weight-bearing radiographs in combination with magnetic resonance imaging would have allowed for a more precise evaluation of loading axes, facilitating measurement of their variation and correlation with the degree of pathology present. Future studies could explore the use of MRI machines capable of maintaining orthostatic positioning during image acquisition.

Finally, no anamnestic information regarding the patients' lifestyle was collected and this could potentially have introduced some confounding factors. Our data need further study, although this work involved a substantial number of patients.

## Conclusions

In conclusion, the literature associates medial longitudinal plantar arch overload with TTP and SLC injuries. Our data are more in line with the theory that considers TTP dysfunction secondary to SLC injury, which in turn is due to internal plantar arch overload with increased calcaneal pronation angle.

Furthermore, our data demonstrate a correlation between increased heel pronation angle and the occurrence of effusion in the talo-calcaneal joint space, a relationship that is also evident with the onset of talar bone oedema.

The analysis of the collected data, evaluating the distribution of pathological findings using ROC curves, allowed us to calculate an average pronation angle value beyond which pathological findings are more likely to be observed.

The calculated cut-off value (resulting from the average of the values specific for each of the binary variables) is approximately 24° (CI 23.9–25.2).

We can conclude that all patients with a pronation angle greater than 24° are predisposed to have medial longitudinal plantar arch overload and that this overload may consequentially cause SLC injury and PTT dysfunction.

## Abbreviations

SLC: Spring ligament complex
CPA: Calcaneal pronation angle
SCSF: Scapho-calcaneal space free fluid
UML: Upper-medial ligament
THE: Talus head oedema
PTT: Posterior tibial tendon
PFE: Plantar fascia enthesopathy
AAFD: Acquired adult flatfoot deformity
